# Does strategic coupling of TNCs affect their exit from host regions: Empirical analysis of the exit of Taiwanese enterprises from mainland China

**DOI:** 10.1371/journal.pone.0304254

**Published:** 2024-05-24

**Authors:** Jindong Chen, Jiping Sun, Suqiong Wei, Xiaojun You

**Affiliations:** School of Geographical Sciences, Carbon Neutrality and Future Technologies, Fujian Normal University, Fuzhou, China; Public Library of Science, UNITED KINGDOM

## Abstract

This study aims to investigate the temporal and spatial attributes of the exit of Taiwanese enterprises from mainland China between 2001 and 2021, by applying enterprise database data. Furthermore, the influence of strategic coupling on Taiwanese enterprises’ exit from mainland China was also investigated. The following are the key findings: The spatial distribution pattern of the exit rate of Taiwanese enterprises in mainland China varied at different phases. In contrast, the inland regions of the country’s central and western zones, which are characterized by comparatively less developed economies, maintained consistently high exit rates, whereas the eastern coastal region retained a low exit rate. Particularly, the relationship between Taiwanese enterprises and the invested areas changed from Captive coupling to Cooperative coupling and subsequently to Absorptive Coupling. Similarly, the coupling modes significantly influenced the exit of Taiwanese enterprises from mainland China. Moreover, the COVID-19 pandemic has contributed to the backward connection of Taiwanese corporations, which have become more reliant on the mainland China market and local suppliers than earlier. Taiwan-favoring policies and the regional innovation environment have consequently emerged as the primary locational advantages for retaining Taiwanese enterprises in the aftermath of the global financial crisis. Therefore, the aforementioned factors may help to reduce the Taiwanese enterprises’ exit from mainland China and possess valuable policy implications for Taiwan investment zones in mainland China.

## Introduction

There has been an ongoing interest among policymakers and academics in examining the factors that influence foreign direct investment (FDI) practices. Meanwhile, the regional perspective of economic geography helps to comprehend the geographical distribution, entry, and exit of FDI in the host countries [1]. However, research examining the exit of transnational corporations (TNCs) after entrance into the host country should place significant emphasis on their external surroundings, specifically the context of competition in various regions of a country with regard to attracting and retaining FDI [2]. Likewise, understanding the strategic management of TNCs is essential because FDI targets strategic assets and market efficacy. Moreover, TNCs decide to withdraw from their host nations and reallocate their assets to other economies for investment purposes when the external circumstances fail to align with their strategic requirements [3]. Meanwhile, both regional and strategic management perspectives of the research related to the exit of TNCs ignore that the investment behavior of TNCs in the host country dynamically corresponds to the elements of the invested region. Concurrently, the existence or absence of TNC development in the host country was determined through collaborative efforts with regional actors and the environment.

Since the exit of TNCs is associated with the entry and expansion behavior of an enterprise, it cannot be understood from a single perspective. Therefore, diverse perspectives must be interconnected [4]. Competitive market pressure influences dominant firms, intermediate product suppliers, and final producers of finished products and services, thereby affecting the geographical configuration of production networks. Specifically, the GPN (Global Production Network) strategy emphasizes the specific roles and numerous interactions of various business and non-business entities within the global production network [5–7]. Consequently, the key concept-strategic coupling combines the regional perspective with the corporate strategic decision-making perspective, which is highly applicable to explain the exit of TNCs in the host country [8, 9].

China, as a developing country, has demonstrated a wide range of economic, political, and geographical variations since implementing its reform and opening up policies. Specifically, TDI is a substantial contributor to foreign investment in mainland China, representing 5.4% of the total FDI in 2021. Accordingly, the "Taiwan Compatriots Investment Protection Law of the People’s Republic of China" was enacted by the Standing Committee of the National People’s Congress to protect and encourage investments from Taiwan compatriots and support economic development on both sides of the Taiwan Straits. This law applies to Taiwanese compatriots when the law does not stipulate. The investment of Taiwanese nationals is governed by other relevant Chinese laws and administrative regulations; their application must adhere to the stipulations therein. Consistent with this, TDI in mainland China serves as an essential strategy for Taiwanese companies to strengthen their overseas market presence and enhance their global competitiveness. Taiwanese investment in mainland China is characterized by unique attributes resulting from the unique political context that exists on both sides of the Taiwan Strait. In addition to exhibiting characteristics typical of overseas investments, Taiwan also demonstrates intricate fluctuations due to its role as an interface connecting domestic and international companies for the "global localization" of investment and production in mainland China [10]. Thus, analyzing the influencing factors and exit status of TDI in mainland China not only provides valuable insights for policymaking but also enables scholars to enhance their comprehension of foreign capital development patterns within the socialist market economy with Chinese characteristics. Additionally, this analysis also presents a special case and supplement of FDI research by highlighting a unique regional relationship and a distinct political context; thereby exerting a strong theoretical significance. This study used strategic coupling as the essential variable from the perspective of relational economic geography, presenting the dual perspective of corporate and external strategy regarding the exit behavior of TNCs, and conducted a quantitative analysis of the impact of strategic coupling on the exit of Taiwanese enterprises in mainland China.

This paper was organized into 6 different sections. Drawing on the core concept of GPN—strategic coupling. The subsequent Section constructed an analytical framework for decoupling multinational corporations from invested regions. The third section of this study presented the methodologies used for data collecting and analysis. The subsequent two empirical sections provided a detailed analysis of the regional and temporal patterns regarding the entry and exit of Taiwanese firms in mainland China, as well as the factors that influence the exit of Taiwanese enterprises from mainland China. Lastly, key study findings and theoretical implications are discussed in the conclusion.

## Literature review and theoretical framework

### Strategic coupling and FDI

GPN describes regional development, particularly as a "strategic coupling" between "regional assets" and "strategic needs of global industries". Moreover, the GPN clarifies the interface mechanism between TNCs and cities/regions through the interaction of various corporate and non-corporate participants; thus, highlighting the geographically sensitive and “territory-based” processes of TNCs embedded in the regional economy [8]. Accordingly, GPN is defined as a worldwide network comprising influential TNCs that control and coordinate a sizable number of strategic partners, overseas affiliates, non-enterprise institutions, and primary consumers [9]. TNCs pursue advantageous couplings in the region following their enterprise strategy, to optimize value, while participants within the production network respond by making strategic decisions. Therefore, regional policymakers seek to integrate GPNs and accelerate local economic development [11].

Yeung proposed that the strategic coupling model can be determined by the control dependence and power relationship between multinational companies and regions. The strategic coupling model can be further defined by the bargaining power of enterprises between the two parties. The coupling mode can be categorized as either functional coupling, structural coupling, or indigenous coupling [12]. Afterward, Liu concluded that Yeung’s three coupling types lack a logical connection and suggested the reconstruction of the strategic coupling framework analysis using the spatial variables of "locational advantages" and "spatial stickiness". Furthermore, the strategic coupling mode can be classified into Captive Coupling, Absorptive Coupling, and Reciprocal Coupling [13]. Meanwhile, the variations in coupling modes explain the transfer [14] and transformation [15] of FDI. Similarly, the GPN methodology considers TNCs as the primary coordinator. However, this paper emphasizes the exit behavior of FDI. Government authorities are reported to be motivated to introduce strategic coupling and FDI, to promote economic growth, seize foreign market opportunities, and assess the spillover effects from MNCs. However, MNCs actively pursue strategic coupling activities to maximize profitability as profit-driven economic entities. This study integrates the two aforementioned perspectives to clarify the relationship between the subjects in the coupling process, considering that TNCs may decide to exit immediately in response to negative shocks.

### Exit: Decoupling of TNCs and the invested regions

**Fig 1:**
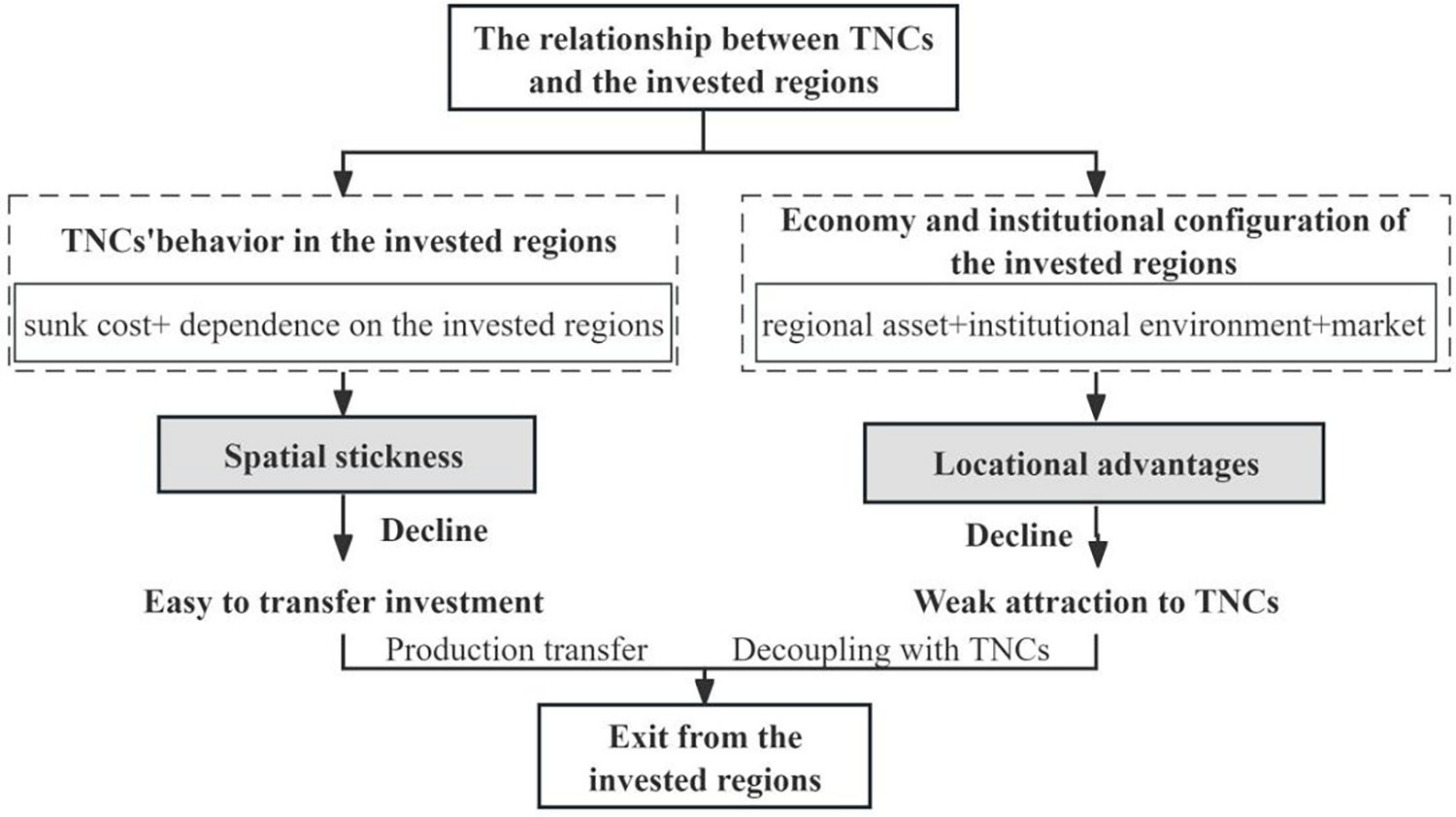
Decoupling of TNCs and regions. Source: Drawn by the authors.

The entry, expansion, and exit are considered the three basic stages of an enterprise life cycle. These three behaviors are interdependent, since the entry, dynamic development, and exit practices of the enterprise occur in a long-term equilibrium state [16]. Typically, the exit of TNCs commonly refers to the cessation of their business operations in the invested region and the withdrawal from that market. The overseas expansion of TNCs is characterized as a socialization and networking process from the perspective of strategic coupling. Specifically, Taiwanese enterprises in mainland China are not TNCs, but rather cross-border enterprises. However, this research paper suggests a universal theoretical framework because of the similarities in their development process with TNCs. The empirical analysis section provides information about the specific attributes of Taiwanese enterprises, emphasizing the interplay and interdependence among customers, employees, enterprises, and regional institutions in economic decision-making, thereby establishing valuable relationship assets. Furthermore, the aforementioned factors elucidate the aggregation of corporate locations as well as subsequent local and regional development [17]. Negative effects of strategic coupling include conflicts, frictions, and even ruptures between the two sides of the coupling due to the significant disparities in interests between TNCs and regions; this can result in decoupling and recoupling [18]. This study used two variables of Liu including strategic coupling-locational advantages and spatial stickiness [13], to describe the entry and exit behavior of TNCs (Fig 1).

Failure to achieve anticipated corporate performance and necessary strategic restructuring leads to the exit of TNCs. Therefore, transnational companies must employ a variety of strategies to adapt to the inherent uncertainties and risks of international practices [19]. However, the capacity of TNCs to operate independently is compromised by the sunk costs associated with establishing overseas production. As the sunk cost of TNCs increases, spatial displacement becomes more challenging due to spatial stickiness. Apart from the sunk costs associated with the production establishment, larger regions tend to have a higher demand for regional resources at the local level and a more complex production organization of TNCs, resulting in increased stickiness. This often results in the development of industrial clusters. The dependence of production methods on regionally scaled systems, talents, and business environments is another manifestation of spatial stickiness. Furthermore, the spatial stickiness of enterprises at the national and even larger scale is based on their reliance on market area, trade barriers, and national macro-political environment. All of the aforementioned factors are associated with spatial stickiness [13].

Specifically, spatial stickiness is more concerned with the individual investment behavior of TNCs. Furthermore, the regional, institutional, and economic configuration showed a high correlation with the entry and exit of TNCs, confirming the earlier stated locational benefits. Superior regional elements allow TNCs to access the local required resources to operate and combine them with their internal resources, to develop competitive relationship assets [20, 21]. Similarly, regional conditions are essential for market-seeking transnational corporations to enter and exit foreign markets [22]. Additionally, government policy also serves as a significant market coordination mechanism that either replaces or complements the market coordination [23]. Particularly, local governments attempt to maximize the popularity of regional markets to TNCs and ensure strategic coupling by minimizing the regulatory barriers for MNCs to facilitate their entrance to the local market. Regulatory bodies realize this initiative by regulating market practices and industrial development following their strategic requirements [24]. Moreover, due to the asymmetry of power between transnational corporations and host countries/regions, TNCs provide a variety of investment location alternatives that are influenced by several factors, including the local environment, local network, and firm-specific strategic considerations [25]. The types of strategic coupling and characteristics of different FDI investment types are shown in Table 1 [26, 27].

**Table 1:** FDI types and transnational corporations—Host region strategic coupling types.

	Absorptive coupling	Reciprocal coupling	Captive coupling
Predominant type of inward FDI	Horizontal	Mixed	Vertical
Coupling motivation	Market or other requirements	Mixed	Efficiency or resources
Degree of foreign ownership and control	Low	Medium	High
Foreign-local firms supply relations	Partnership	Mixed	Dependency
The embeddedness of transnational corporations	High	Medium	Low
Predominant FDI linkages	Developmental	Mixed	Dependent
Value capture	High	Medium	Low
Type of invested region	Developed regions	Regions with rapid economic development	Less developed regions
Regional advantage	High	Medium	Low
Spatial stickiness	High	Medium	Low
Exit (decoupling) possibility	Low	Medium	High

The "global localization" attribute is evident in mainland Chinese Taiwanese investment, serving as a connection between local regions and the global marketplace, with Taiwan occupying a central position. Primarily, Taiwanese businesses integrate into the global production network *via* the establishment of their own "secondary global production network" through investment in mainland China; thus, they can participate in the global labor division. The term "secondary production network" refers to the cross-border production network and spatial organization of Taiwanese-funded enterprises in host zones. Generally, these enterprises typically hold a relatively inferior function in the global labor division. They are involved in activities such as assembly, processing, or primary production, and work effectively with dominant global companies within the GPN. Therefore, the position of Taiwanese enterprises in the global production network needs to be elaborated.

## Data and methodology

The time period range in this study was typically from 2001 to 2021. The data on Taiwanese enterprises was obtained from the largest enterprise information query website in China (Qcc:https://www.qcc.com/) which includes the detailed address, establishment time, exit time, enterprise size, and other enterprise-related information. Furthermore, the field of study data was integrated with the ’Mainland Taiwanese Enterprise Directory (2010 Edition)’ for proofreading. Therefore, the sample that experienced significant information loss was excluded. Hence, the data of a total of 61081 Taiwanese firms was obtained in this paper. Significantly, corporations have extensive data coverage that holds a particular level of authority due to their years of operation and accumulation of data. Using the Conditional filtering Settings of PYTHON, it is possible to verify that the data obtained from the web crawl specifically corresponds to the research requirements. The acquired data can be proofread and supplemented with the Mainland Taiwanese Enterprise Directory (2010 Edition), to further ensure the data accuracy.

Moreover, the data on strategic coupling variables was derived from the "Survey and Analysis of Investment Business Operation in Mainland China" published by the Taiwan Investment Review Commission as well as the Statistical Yearbooks of each province throughout the study. This article used the exit and entry rates of Taiwanese firms in mainland China to demonstrate the survival status of Taiwanese enterprises in the region. Thereafter, the data from various firms was combined at the provincial level to determine the total number of new and present enterprises at the end of each phase. The entry rate refers to the ratio of the number of newly established enterprises to the number of surviving enterprises. On the other hand, the exit rate is the proportion of the number of exiting enterprises to the number of surviving enterprises.

The quantitative measurement of strategic coupling is the primary challenge of this study. Among the current studies, only Liu measured the degree of strategic integration between regional and global production networks by employing the three dimensions of export-led, technology-led, and production-led [28]. However, following one of the definitions of GPN—embeddedness [5, 29], this paper advocates that the relevant research indicators concerning the relationship between TNCs and invested regions may also be applied to quantify spatial stickiness. To quantify the critical variables of the strategic coupling between Taiwanese enterprises and invested regions (Table 2), the indicators were selected from the two dimensions of location advantage and spatial stickiness, following the aforementioned discussion and the availability of the variable data.

**Table 2:** Variable description.

	Variables	Specific Indicators	Measurement
**Explained variable**	Exit degree of Taiwanese enterprises	Exit rate of Taiwanese enterprises	Number of exit Taiwanese enterprises divided by the number of existing Taiwanese enterprises
**Explanatory variables (1)**	Regional advantage	Regional innovation environment	R&D personnel full-time equivalent
		Regional market size	GDP
		Regional infrastructure	Transport highway mileage (average)
		Regional institutional advantages	The number of Taiwan-favoring policies and national Taiwan investment zones
**Explanatory variables (2)**	Spatial stickiness of Taiwanese enterprises	Investment amount	Annual investment amount of Taiwanese enterprises
		Technical cooperation with the regional institute	Taiwanese enterprises having technical cooperation with mainland China institutions divided by all Taiwanese enterprises
		Institutional matching	The preferential policy-related weighted ratio of Taiwanese enterprise investments in mainland China
		Industrial chain stickiness	Number of Taiwanese enterprises with raw materials, parts, and semi-finished products from the mainland divided by the number of all Taiwanese enterprises
		Market stickiness	Domestic income share of Taiwanese enterprises
		Local labor stickiness	The proportion of mainland China employees in Taiwanese enterprises
**Control variables**	-	Taiwanese enterprises concentration	Total number of Taiwanese enterprises since 1990
	Environmental mutation	External relationship	Political interaction index between mainland China and Taiwan [30]
		COVID-19	2020, 2021 = 1, Others = 0

Using the strategic coupling explanatory variables in Table 2, this study constructed a regression model to examine the correlation between the strategic coupling of Taiwanese enterprises in mainland China and their exit behavior. Similarly, the environmental mutation variables and the degree of aggregation among Taiwanese enterprises were used as control variables. The lag effect of explanatory factors on other explanatory variables causes certain variables to be lagged.

## Spatial and temporal characteristics of Taiwanese enterprise’s exit from mainland China

**Fig 2:**
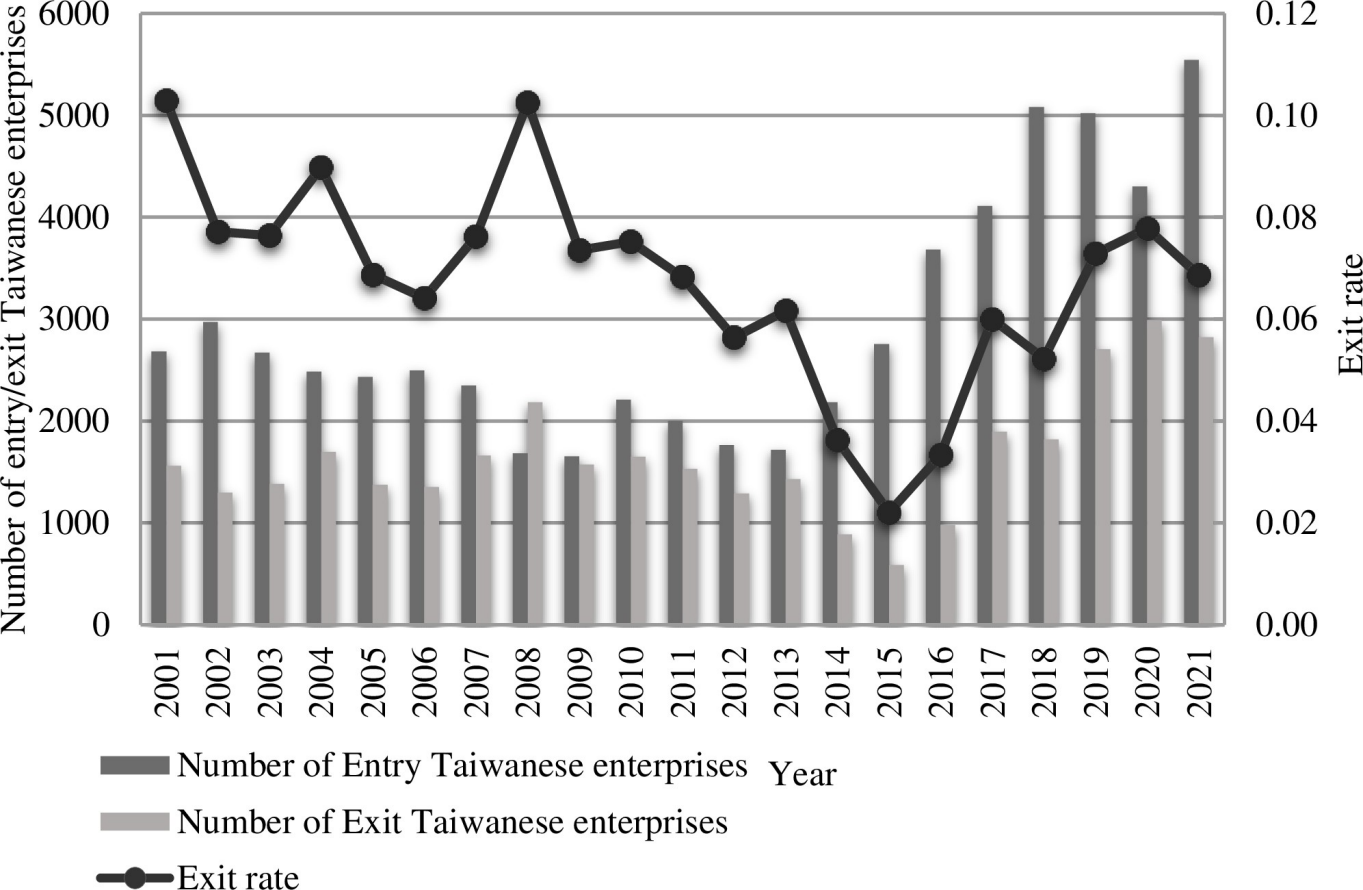
Entry and exit of Taiwanese enterprises in mainland China from 2001 to 2021. Source: Drawn by the authors.

Taiwanese investment and cross-strait trade in mainland China experienced significant growth in the mid-1980s. This was primarily due to the economic reforms implemented in mainland China and the progressive relaxation of restrictions on cross-strait economic interaction. However, there was an increase in Taiwanese direct investment in mainland China after the year 2000 [31]. Furthermore, the instability of cross-strait ties, specifically the implementation of the "Southward Policy" in 1993, exerted a negative impact on Taiwanese investment in mainland China. A total of 18,727 Taiwanese firms have been invested in mainland China before 2000. Afterward, 4,691 were determined to be invalid and a total of 14,036 remained by 2000. Furthermore, the Taiwan authorities have relaxed the trade restrictions on Taiwanese direct investment in mainland China since 2000, because of the improved relations between Taiwan and mainland China. The withdrawal of Taiwanese firms from Mainland China can be categorized into three distinct stages (Fig 2): 2001–2007, 2008–2013, and 2014–2021. Taiwanese investment in the mainland experienced consistent development during the initial phase. The proposed expansion was influenced by several events, including the accession of both sides of the Taiwan Strait to the World Trade Organization in 2001, the promulgation of the Interim Measures for the Administration of Taiwan Compatriots’ Investment Enterprises Association by the mainland in 2003, and the issuance of the Shared Vision for the Peaceful Development of Cross-Strait Relations in 2005. The number of entering enterprises was recorded to be significantly greater during this phase than that of exiting enterprises, while the number of existing enterprises gradually increased with time. In the second stage, significant progress was observed in cross-strait relations, including the official launch of direct flights across the Taiwan Strait in 2008, the signing of the Cross-Strait Investment Protection and Promotion Agreement in 2012, and the Cross-Strait Economic Cooperation Framework Agreement (ECFA) in 2010. However, investment activities of Taiwanese corporations on the mainland have been restricted due to the global financial crisis. Therefore, the number of exiting enterprises approached that of entering enterprises during this phase. While in 2008, the number of exiting enterprises even exceeded that of entering enterprises. Therefore, the development of the existing enterprises becomes slow and almost stagnant. The third phase of cross-strait economic and trade interactions encountered major challenges, as cross-strait relations deteriorated after 2016; consequently, international trade frictions increased in 2018; and the novel strain of COVID-19 pneumonia emerged in 2020. Despite the global economic downturn, mainland China has actively promoted economic collaboration across the Taiwan Strait, implementing "11 measures" to assist Taiwanese enterprises in overcoming the detrimental consequences of the COVID-19 pandemic, and "31 measures" and "26 measures" to facilitate cross-strait economic and cultural exchanges. Currently, a large number of Taiwanese enterprises have been established in mainland China. While the third stage observed satisfactory growth in the number of exiting Taiwanese enterprises, the number of existing enterprises also expanded at a faster rate than in the preceding two phases. There was a 14% decrease in the establishment of new Taiwanese enterprises during the initial year (2020) of the COVID-19 pandemic in mainland China. Furthermore, there was no significant disparity in the number of enterprises’ exits before and after the pandemic. In the second year (2021), the number of new Taiwanese firms in mainland China continued to increase, while the number of exits remained the same. These findings suggest that the trade exchanges and cross-strait economies have generally maintained their growth rates despite adverse factors such as the COVID-19 pandemic.

The entry and exit rates of Taiwanese enterprises in the provinces and autonomous regions of the mainland are displayed on the map following the three phases of withdrawal, as shown in Table 3. Specifically, the Pearson correlation analysis on the entry- and exit rates of the provinces in the three stages respectively revealed the following results of the correlation analysis: -0.3633*, -0.5694**, and 0.2310*. The observed finding was different from that previously reported literature showing the positive correlation between the entry and exit rate of Chinese foreign-invested firms [1]. Even though Taiwanese investment signifies foreign investment in mainland China, the spatial evolution law governing general foreign investment behavior does not apply to the development dynamics of these enterprises due to the unique political contexts of China and Taiwan. Furthermore, as China’s opening-up policy was further strengthened from 2001 to 2007, investment from Taiwan started to shift towards inland regions, while the development of foreign capital in the eastern coastal zones tended to reach a mature stage. Enhanced activity in the entrance of Taiwanese investments occurred following the "Western development" policy, resulting in an increased entry rate. Even though the Western region initially lacks a solid foundation for the growth of Taiwan investment. However, preceding Taiwanese enterprises were devoid of adequate intrinsic motivation, and exit behavior remained elevated due to the lack of sound policy, traffic location, economic development, and certain other factors in the central region. Furthermore, between 2008 and 2013, the rate of entry of Taiwanese capital into each province was lower than in the other two stages, whereas the exit rate was higher than in the other two stages. Conversely, the regions with high entry rates were concentrated in the developed western and coastal provinces; while the regions with active exit were primarily located in the northern inland provinces. Moreover, from 2014 to 2021, entry-level Taiwanese enterprises rebounded nationwide from the previous stage, with the southwest and south-central provinces having the highest entry rates. Moreover, exit behavior was decreased substantially from the previous spatial scope stage. Particularly, the high exit rate was concentrated in two economically backward inland provinces, Ningxia and Jiangxi.

**Table 3:** Provincial distribution of entry and exit Taiwanese enterprises in mainland China from 200l to 202l.

Region	Province	Exit rate			Entry rate		
		2001–2007	2008–2013	2014–2021	2001–2007	2008–2013	2014–2021
**Northeastern region**	**Liaoning**	0.5087	0.4472	0.3496	0.3946	0.1130	0.3291
	**Jilin**	0.1563	0.3455	0.2500	0.0294	0.5490	0.4000
	**Heilongjiang**	0.3105	0.4276	0.3419	0.3316	0.1123	0.4000
**Easter region**	**Bejing**	0.0700	0.3173	0.2801	0.6304	0.5778	0.4344
	**Tianjin**	0.1071	0.5613	0.2595	0.4268	0.1925	0.3804
	**Hebei**	0.3346	0.5067	0.2109	0.4926	0.1419	0.4304
	**Shanghai**	0.1342	0.1317	0.1872	0.6856	0.5074	0.4355
	**Jiangsu**	0.3062	0.3563	0.2394	0.6397	0.3710	0.3892
	**Zhejiang**	0.3018	0.2281	0.3057	0.5813	0.2325	0.4132
	**Fujian**	0.2496	0.2474	0.2737	0.5025	0.4023	0.4905
	**Shandong**	0.4813	0.3677	0.2703	0.5331	0.1450	0.4054
	**Guangdong**	0.2856	0.2584	0.2734	0.5543	0.3049	0.4275
	**Hainan**	0.4741	0.3956	0.2894	0.2023	0.0472	0.4199
**Central region**	**Shanxi**	0.1951	0.4773	0.0714	0.4390	0.2245	0.4388
	**Anhui**	0.5076	0.4298	0.2057	0.5271	0.2038	0.4617
	**Jiangxi**	0.3128	0.3329	0.5105	0.7113	0.4130	0.4650
	**Henan**	0.5488	0.4537	0.1920	0.3253	0.1298	0.3844
	**Hubei**	0.4766	0.3448	0.2442	0.4133	0.1865	0.4160
	**Hunan**	0.5517	0.3750	0.3583	0.5052	0.1544	0.5072
**Western region**	**Inner Mongolia**	0.6346	0.6087	0.3077	0.3269	0.0476	0.4857
	**Guangxi**	0.0849	0.2428	0.2255	0.5634	0.5062	0.4706
	**Chongqing**	0.4660	0.1584	0.3626	0.4215	0.2616	0.4568
	**Sichuan**	0.2697	0.3216	0.3130	0.4474	0.3728	0.5116
	**Guizhou**	0.5781	0.1875	0.2179	0.4063	0.2658	0.5018
	**Yunnan**	0.3746	0.4245	0.3629	0.2680	0.1618	0.4600
	**Shaanxi**	0.2453	0.2899	0.3911	0.4151	0.3267	0.4588
	**Gansu**	0.3478	0.4412	0.3793	0.3261	0.0870	0.3571
	**Qinghai**	0.2000	0.6000	0.0833	1.0000	0.2500	0.4762
	**Ningxia**	0.5625	0.7500	0.5000	0.2917	0.0612	0.6000
	**Xinjiang**	0.1538	0.2222	0.3636	0.6923	0.1795	0.4750

Table 4 presents the global Moran index for the entry and exit of Taiwan-funded enterprises in mainland China over three distinct time periods: 2001–2007, 2008–2013, and 2014–2021. The P-value for each period was smaller than that of the commonly used significance level of 0.05, indicating a significant spatial autocorrelation in the distribution of Taiwanese enterprises entering and exiting mainland China at a 95% confidence level. This conclusion was further supported by the high Z-value, which indicated that the entry and exit behaviors of Taiwanese businessmen in space showed a significant trend toward either clustering or dispersion. It is worth noting that all the I values were greater than zero, indicating a distinct clustering trend in the spatial distribution of Taiwanese businessmen’s entry and exit behaviors, rather than a random distribution.

**Table 4:** Global Moran’s I index for the entry and exit of mainland Taiwanese-owned enterprises from 2001 to 2021.

	**2001–2007**		**2008–2013**		**2014–2021**	
	**Entry rate**	**Exit rate**	**Entry rate**	**Exit rate**	**Entry rate**	**Exit rate**
**Moran’I**	0.386	0.413	0.254	0.242	0.285	0.357
**P-value**	0.000	0.000	0.001	0.007	0.003	0.000
**Z-score**	3.475	3.581	3.221	2.462	2.800	3.394

Tables 5 and 6 present the local spatial autocorrelation of entry and exit rates for mainland and Taiwan enterprises, which were divided into four types: high-high (HH), low-high (LH), low-low (LL), and high-low (HL). In HH regions, high values were surrounded by other high-value regions; in LH regions, low values were surrounded by high-value regions; in LL regions, low values were surrounded by other low-value regions; and in HL regions, high values were surrounded by low-value regions. Entry and exit rates in both H-H and L-L regions were highly consistent and varied gradually over time. The H-H type region included Shanghai, Zhejiang, Fujian, Jiangsu, Guangdong, and Shandong, all of which are located in the eastern coastal regions. Meanwhile, the L-L type region was comprised of Henan, Hebei, Beijing, Hubei, Hunan, Chongqing, Guizhou, Guangxi, Sichuan, Yunnan, Jilin, Heilongjiang, Shanxi, Gansu, and Xinjiang, all of which are located inland or in economically underdeveloped regions in the west. The L-H region comprised neighboring economically developed provinces, including Anhui, Hainan, Jiangxi, and Guangxi, which probably experienced their development momentum while also being influenced by these neighboring regions.

**Table 5:** LISA distribution of the number of Taiwanese enterprises entering different phases from 2001 to 2021.

	2001–2007	2008–2013	2014–2021
**HH**	Shanghai, Zhejiang, Fujian, Jiangsu, Guangdong, Shandong	Shanghai, Zhejiang, Fujian, Jiangsu, Guangdong, Shandong	Shanghai, Zhejiang, Fujian, Jiangsu, Guangdong
**LH**	Anhui, Hainan, Jiangxi, Guangxi, Hunan	Anhui, Hainan, Jiangxi, Guangxi, Hunan	Anhui, Hunan, Jiangxi, Guangxi, Shandong
**LL**	Henan, Hebei, Beijing, Hubei, Chongqing, Guizhou, Guangxi, Sichuan, Yunnan, Jilin, Heilongjiang, Shanxi, Gansu, Xinjiang, Tianjin	Henan, Hebei, Beijing, Hubei, Hunan, Chongqing, Guizhou, Guangxi, Sichuan, Yunnan, Jilin, Heilongjiang, Liaoning, Shanxi, Gansu, Xinjiang	Henan, Hebei, Beijing, Tianjin, Hubei, Hunan, Chongqing, Guizhou, Guangxi, Sichuan, Yunnan, Jilin, Heilongjiang, Liaoning, Shanxi, Gansu, Xinjiang
**HL**	—	—	—

**Table 6:** LISA distribution of the number of Taiwanese enterprises entering different phases from 2001 to 2021.

	2001–2007	2008–2013	2014–2021
**HH**	Shanghai, Zhejiang, Fujian, Jiangsu, Guangdong, Shandong	Shanghai, Zhejiang, Fujian, Jiangsu, Guangdong, Shandong	Shanghai, Zhejiang, Fujian, Jiangsu, Guangdong
**LH**	Anhui, Hainan, Jiangxi, Guangxi	Anhui, Hainan, Jiangxi, Guangxi	Anhui, Hunan, Jiangxi, Guangxi, Shandong
**LL**	Henan, Hebei, Beijing, Hubei, Hunan, Chongqing, Guizhou, Guangxi, Sichuan, Yunnan, Jilin, Heilongjiang, Shanxi, Gansu, Xinjiang, Tianjin	Henan, Hebei, Beijing, Hubei, Hunan, Chongqing, Guizhou, Guangxi, Sichuan, Yunnan, Jilin, Heilongjiang, Liaoning, Shanxi, Gansu, Xinjiang	Henan, Hebei, Beijing, Tianjin, Hubei, Hunan, Chongqing, Guizhou, Guangxi, Sichuan, Yunnan, Jilin, Heilongjiang, Liaoning, Shanxi, Gansu, Xinjiang
**HL**	Liaoning	Tianjin	—

## The influencing factors of Taiwanese enterprises’ exit from mainland China

This study conducts regression analysis on samples from three distinct research periods (2001–2007, 2008–2013, 2014–2021). The purpose is to examine the relationship between Taiwanese enterprises and regions, taking into account the time-sensitive nature of this coupling and its sensitivity to dynamic changes in the regional background, such as the emergence of high-tech enterprises and market growth [27]. The regression results are displayed in Table 7. The R^2^ values for the three research periods are 0.6496, 0.6330, and 0.7337, respectively. This suggests that when considering the years 2014 to 2021, the strategic coupling behavior of Taiwanese enterprises has a more pronounced impact on their exit. Moreover, the regional infrastructure and market size factors, which are focused on the local location’s advantageous variables, have a significant detrimental effect on the exit of Taiwanese enterprises from 2001 to 2007. During the second phase of Taiwanese enterprise investment in mainland China, the focus of investment is mostly on the spatial stickiness variable, which exhibits a strong negative association with the exit rate of Taiwanese enterprises. Moreover, the size of the local market is focused on the advantageous factor of the local location. Afterward, the stickiness of the labor force in the first two stages has a notably favorable impact on the exit of Taiwanese enterprises. This suggests that Taiwanese enterprises prioritize low local labor costs, despite their initial reliance on the local labor supply during the early stages of their investments in mainland China. However, this vertical investment is not viable over an extended period. The presence of favorable institutional factors and a conducive regional innovation environment, together with an abundance of advantageous local resources exert a negative effect on the exit of Taiwanese enterprises in mainland China from 2014 to 2021. The spatial stickiness variables, including investment scale, industrial chain stickiness, and market stickiness, have a considerable negative impact on the exit of Taiwanese enterprises in mainland China. Similarly, the two factors of technological stickiness and institutional matching have shown to have a negligible effect over the entire research period. This indicates that these two factors do not play a role in the strategic coupling process of Taiwanese investment enterprises on their exit behavior in mainland China. The COVID-19 pandemic and the concentration of Taiwanese enterprises in Taiwan from 2014 to 2021 had a favorable impact as control variables. However, the external relationship variable does not play a substantial role in the process of the strategic coupling of Taiwanese enterprises on their exit from mainland China.

**Table 7:** Multiple linear regression.

		2001–2007	2008–2013	2014–2021
	**Constant**	1.4127** (3.4714)	0.9727** (3.3229)	-0.5169** (-3.2448)
**Regional advantage**	Regional innovation environment	0.0309 (1.5314)	0.0312 (1.7373)	-0.0213* (-2.6257)
	Regional market size	-0.0845* (-2.4859)	-0.1089** (-2.7062)	-0.0002 (-0.6361)
	Regional infrastructure	-0.1531** (-3.3995)	-0.0150 (-1.0402)	0.0001 (0.2739)
	Regional institutional advantages	0.0005 (0.4787)	0.0005 (0.7138)	-0.0093* (-2.0176)
**Spatial stickiness of Taiwanese enterprises**	Investment amount	-0.0171 (-1.4382)	-0.0130* (-2.0788)	-0.0579** (-4.4651)
	Technical Cooperation with the Regional Institute	0.0005 (0.7901))	-0.0020 (-0.0968)	-0.0003 (-1.2201)
	Institutional matching	0.0007 (0.3211)	-0.0008 (-0.4037)	-0.0003 (-1.0980)
	Industrial chain stickiness	-0.0006 (-1.3247)	0.0005 (1.1787)	-0.0064* (-2.4939)
	Market stickiness	-0.0171 (-1.4382)	-0.0006 (-1.1851)	-0.1718* (-2.3779)
	Local labor stickiness	0.1551** (4.7373)	0.1849** (6.2571)	0.0005 (0.6834)
**Control variable**	Taiwanese enterprises concentration	0.0000* (2.5025)	0.0000*(2.3326)	0.0012* (2.4636)
	External relationship	-0.0124 (-0.8350)	0.1279 (0.199)	-0.0016 (-0.9984)
	COVID-19	-	-	0.0131* (2.3818)
	R^2^	0.7457	0.7431	0.8248
	Adjusted R^2^	0.6496	0.6330	0.7337

Robust standard errors are in parentheses.

***Significant at 1%.

**Significant at 5%.

*Significant at 10%.

This study examines the strategic coupling and exit behavior of Taiwanese investment enterprises in mainland China from 2001 to 2021 using multiple linear regressions. The findings are presented in Table 8. The exceptional infrastructure and substantial regional market size have been highly appealing to Taiwanese enterprises for their ongoing expansion in mainland China between 2001 and 2007. In addition, the labor stickiness stimulates the exit of Taiwanese enterprises in mainland China. Currently, Taiwanese enterprises are mostly driven to invest in mainland China to improve operational efficiency, reduce costs, and avoid the necessity of high-level relationship capital. The majority of enterprises implement a ’two-end out’ strategy, in which Taiwan is responsible for high-value-added activities such as research and development and sales, while the mainland focuses on manufacturing and exports. Currently, Taiwanese enterprises with limited available resources prefer to focus on expanding their presence in the local market of mainland China. Similarly, Taiwanese enterprises prioritize the level of infrastructural development in mainland China. Given the limited market size and bargaining strength of the invested areas, Taiwanese enterprises also focus on these factors. In addition, most industries with low labor costs typically do not have high-value non-production services. These industries primarily focus on producing standardized products for export, such as assembly, mining, or daily service operations in the investment field. This not only reduces the value capture process but also weakens the links between Taiwanese enterprises and local enterprises. Therefore, to expand and improve their financial performance, Taiwanese enterprises find it convenient to sacrifice other participants, such as employees and local corporations. Currently, the strategic relationship between mainland and Taiwanese investment businesses can be characterized as Captive Coupling, where the coupling is based on the benefit of low cost. However, the advantages of location and spatial stickiness are not significant.

**Table 8:** Evolution of strategic coupling model between Taiwanese investment regions and Taiwanese enterprises in mainland China.

Phase	Coupling mode	Regional advantage	Spatial stickiness	Strategic coupling motivation	Value capture	Exit (decoupling) possibility
**2001–2007**	Captive coupling	Low	Low	Efficiency or low-cost resources	Low	High
**2008–2013**	Reciprocal Coupling	Medium	Medium	Efficiency or low-cost resources, markets	Medium	Medium
**2014–2021**	Absorption coupling	High	High	Market, institutional environment, and innovation environment	High	Low

During the period from 2008 to 2013, the size of the regional market played a significant role in determining the advantages of a certain location. In line with this, the initial investment scale of Taiwanese enterprises also started to influence their choice to withdraw from mainland China. Meanwhile, Taiwanese enterprises that had achieved a significant economic size started to generate profits. Therefore, substantial sunk costs increased the stickiness of Taiwanese enterprises on the mainland. Simultaneously, the domestic demand market for Taiwanese companies in mainland China exceeded the cost factor. However, labor cost and production orientation continue to be the primary strategic focus for the development of Taiwanese enterprises in mainland China. Currently, the location advantage has not only improved, but the region also demonstrates a certain level of bargaining strength. Moreover, the strategic coupling between the mainland and Taiwanese enterprises has been transformed into Reciprocal Coupling. The development of Taiwanese enterprises in the mainland primarily focuses on the significant domestic market scale. The strategic coupling between multinational corporations and local corporations is currently deficient due to the limited capabilities of local companies. Furthermore, mainland Chinese corporations have not successfully incorporated Taiwanese companies into their manufacturing networks.

From 2014 to 2021, the locational advantages factors that slowed down the exit of Taiwanese enterprises from mainland China changed from market size to innovation environment and institutional advantages. The regional innovation environment variables, such as the number of full-time equivalent R&D professionals in various regions, indicate the prospective R&D capabilities and the concentration of a skilled labor force in the location of investment. The suggested factors have the potential to enhance the profitability of technology-based TNCs and expand their expertise in high-end technology fields [32]. Currently, the level of R&D ’localization’ has increased as the Taiwanese industry in mainland China has increasingly shifted high-end R&D investment away from the original processing trade. Consequently, the primary factor that has led Taiwanese enterprises to remain rooted in the regional innovation environment. However, the technological connection between these enterprises and the regions does not influence their decision to exit, as they prefer to develop their R&D practices. Taiwanese enterprises were able to maintain operations throughout the COVID-19 crisis due to the successful pandemic control measures and execution of Taiwan-favoring policies in mainland China despite external influences such as Sino-U.S. trade frictions. In mainland China, the spatial stickiness factors of Taiwanese enterprises have increased under external shocks as compared with the previous two stages. In addition to the scale of early investment, there is a shift in the situation of ’Taiwan orders and mainland China shipping’, allowing Taiwanese products and services to focus on the domestic market while becoming more dependent on the mainland Chinese market. The pandemic-induced disruption of international connections has prompted Taiwanese enterprises to shift towards localizing the global production chain and strengthening cooperation with domestic suppliers. Therefore, the coupling mode between mainland China and Taiwanese enterprises has changed from Reciprocal Coupling to Absorptive Coupling. Similarly, there have been advancements in terms of spatial stickiness, location benefits, and regional bargaining power. As the degree of localization of business operations in Taiwan has increased, the motivation to migrate abroad has become insufficient.

## Conclusions

Multinational enterprises have made substantial contributions to the economic progress of China. TNCs have emerged as a crucial driving factor in China’s integration into worldwide industrial networks [33]. Previous research on the withdrawal behavior of TNCs has predominantly focused on the perspective of corporate strategic management and the external environment. This research investigates the spatial dynamics of Taiwanese enterprises withdrawing from mainland China using data from an official enterprise data query platform in China. This paper quantitatively estimates two essential variables, strategic coupling-location advantage, and spatial stickiness, to evaluate the reasons driving their exit. This study also examines the mechanism of the regional economy, institutional structure, and strategy management behavior of Taiwanese enterprises in relation to their production relocation.

The main findings of this paper can be summarized as follows: The exit of Taiwanese businesses from mainland China is primarily focused on inland provinces, which contrasts with the entry of Taiwanese enterprises. The results of multiple linear regressions indicate that strategic coupling has a significant influence on the exit of Taiwanese enterprises from mainland China. Similarly, the two dimensions of spatial stickiness and local location advantage of Taiwanese enterprises can effectively reflect the mechanism of strategic coupling on their exit behavior. The interaction between mainland China and Taiwanese enterprises in the 3 research periods can be described as strategic coupling, specifically categorized as Captive Coupling, Reciprocal Coupling, and Absorptive Coupling. Furthermore, the phenomenon of spatial stickiness and the benefits of location advantage continue to increase with time. The loss in local location advantages, such as regional market scale and infrastructure level, led to the withdrawal of Taiwan enterprises in mainland China between 2001 and 2007. From 2008 to 2013, the exit rate of Taiwanese enterprises in the Chinese mainland decreased due to the reduced early investment scale and regional market size of Taiwanese enterprises. During the specified research periods, the exit of Taiwanese enterprises is greatly facilitated due to the local labor stickiness, while the advantages of low labor costs do not contribute to a positive coupling. The strategic coupling relationship between Mainland China and Taiwanese enterprises has undergone significant changes from 2014 to 2021. These changes are primarily characterized by an increase in spatial stickiness and an improvement in locational advantages. Simultaneously, the size of investment, market stickiness, industrial chain stickiness, regional innovation environment, and institutional advantages have facilitated the ongoing establishment of Taiwanese businesses in Mainland China. To summarize, conventional production factors, including the scale of regional markets and the quality of infrastructure, have progressively lost their significance in influencing the location choices of Taiwanese enterprises. Conversely, the significance of institutional advantages and the innovation environment has grown substantially in this context.

This work makes two primary contributions to the current field of literature. Firstly, this study provides a positive and efficient method to quantitatively assess strategic coupling, hence enhancing the quantitative research on global production networks. Furthermore, this study avoids only examining the exit of multinational enterprises from either an internal or external perspective. Instead, it aims to comprehensively understand the geographical and multi-scale aspects of multinational enterprises ‘exit. This research examines the factors that influence the withdrawal of Taiwanese investment in mainland China from a dynamic perspective. It confirms the process of strategic coupling in relation to the differentiation of TDI’s exit. The study findings suggest that the purpose and quality of coupling play a crucial role in understanding the relationship between TDI and regional development in developing economies. Furthermore, the main drivers of TDI have changed from low labor and land costs to factors such as a large domestic market, Taiwan-favoring regulations, the thriving national economy, and the competitiveness of the regional innovation ecosystem. Enterprises now prioritize seizing China’s market potential as a crucial aspect of their business plans, given the global economic downturn in the post-pandemic era. Therefore, policymakers should effectively manage a range of local development resources, in a mutually beneficial partnership with TNCs, and actively explore opportunities to enhance the local economy inside the GPN. To conduct a more comprehensive analysis, this paper should incorporate industry heterogeneity into the study of the exit status of Taiwanese enterprises investing in Mainland China. It should also examine the driving factors and development trends of different industries. This will provide a more thorough understanding of the topic.
